# Supplementary material for: Global hypo-methylation in a proportion of glioblastoma enriched for an astrocytic signature is associated with increased invasion and altered immune landscape
Source: eLife. 2022 Nov 22;11:e77335. doi: 10.7554/eLife.77335 (PMC9681209; doi:10.7554/eLife.77335)
Supplement: Figure 2—source data 1. [file elife-77335-fig2-data1.zip › Figure_2_source_data_1/Figure_2B/homerResults/motif6.similar.html]

motif6

## Information for motif6

C
T
G
A
A
C
T
G
C
T
G
A
C
G
T
A
C
T
A
G
A
C
T
G
C
G
T
A
A
C
T
G
A
G
T
C
A
G
C
T
  
Reverse Opposite:  

C
T
G
A
A
C
T
G
A
G
T
C
A
C
G
T
A
G
T
C
A
G
T
C
A
C
G
T
A
G
C
T
A
G
T
C
A
G
C
T
  

|  |  |
| --- | --- |
| p-value: | 1e-72 |
| log p-value: | -1.678e+02 |
| Information Content per bp: | 1.931 |
| Number of Target Sequences with motif | 94.0 |
| Percentage of Target Sequences with motif | 4.07% |
| Number of Background Sequences with motif | 1.7 |
| Percentage of Background Sequences with motif | 0.48% |
| Average Position of motif in Targets | 102.1 +/- 52.6bp |
| Average Position of motif in Background | 135.7 +/- 15.7bp |
| Strand Bias (log2 ratio + to - strand density) | -0.2 |
| Multiplicity (# of sites on avg that occur together) | 1.04 |
| Motif File: | file (matrix) reverse opposite |

### Similar de novo motifs found

|  |  |  |  |  |  |  |  |
| --- | --- | --- | --- | --- | --- | --- | --- |
| Rank | Match Score | Redundant Motif | P-value | log P-value | % of Targets | % of Background | Motif file |
| 1 | 0.617 | C T A G C G T A A C T G C T A G C G T A A C T G G T A C C G A T A T C G A C G T G T A C A G T C A G T C C A G T A T C G | 1e-44 | -102.862651 | 3.94% | 0.83% | motif file (matrix) |
